# The adaptation of SARS-CoV-2 to humans

**DOI:** 10.1590/0074-02760210127

**Published:** 2022-01-10

**Authors:** Eduardo Tosta

**Affiliations:** 1 Universidade de Brasília, Faculdade de Medicina, Brasília, DF, Brasil

**Keywords:** SAR-CoV-2, adaptive evolution, mutation and variants, escape, immunity

## Abstract

The process of adaptation of severe acute respiratory syndrome coronavirus 2 (SARS-CoV-2) to humans probably had started decades ago, when its ancestor diverged from the bat coronavirus. The adaptive process comprises strategies the virus uses to overcome the respiratory tract defense barriers and replicate and shed in the host cells. These strategies include the impairment of interferon production, hiding immunogenic motifs, avoiding viral RNA detection, manipulating cell autophagy, triggering host cell death, inducing lymphocyte exhaustion and depletion, and finally, mutation and escape from immunity. In addition, SARS-CoV-2 employs strategies to take advantage of host cell resources for its benefits, such as inhibiting the ubiquitin-proteasome system, hijacking mitochondria functions, and usage of enhancing antibodies. It may be anticipated that as the tradeoffs of adaptation progress, the virus destructive burden will gradually subside. Some evidence suggests that SARS-CoV-2 will become part of the human respiratory virome, as had occurred with other coronaviruses, and coevolve with its host.

Together with the social and economic disruption produced by the severe acute respiratory syndrome coronavirus 2 (SARS-CoV-2) pandemic, its impressive death toll conveys the idea that the virus is inflicting a war against humankind. That is not the case. Though destruction is the visible face of the contention, it is not its aim. The contenders are viral particles from one side, and the other, cells and molecules of the infected person. These elements have no volition and, therefore, they cannot display any intention, including causing harm to each other. Instead, they act accordingly to biochemical signals that evolutionary forces have shaped to afford adaptation.

Destruction of the counterpart is harmful to both sides. If the virus destroys its host, it is destroyed as well, and if the host causes early elimination of the virus, immunity is not achieved. The progenitors of both SARS-CoV-2 and humans have developed, over evolutionary times, mechanisms whose ultimate aim is to live in equilibrium and evolve, which implies coadaptation and coevolution.^1^ ^,^ ^2^ ^,^ ^3^ Yet, before an adequate adaptation level is reached, destruction is inevitable. Indeed, the SARS-CoV-2 pandemic has caused an enormous amount of deaths worldwide, and a massive amount of viruses has been destroyed by those who recovered from the infection.

The Greek philosopher Heraclitus observed about 2500 years ago that ‘there is nothing permanent except change’. Indeed, all living beings and the physical landscape where they live are constantly changing, and adaptation is a major force behind this change. Adaptation depends on tradeoffs between organisms aiming to improve mutual fitness and survival. It involves two primary mechanisms, selective pressure and mutation, both operating on SARS-CoV-2. Gene sequences of SARS-CoV-2 isolated from different geographic areas confirm that genetic changes of the virus are continuously arising, which ultimately promote adaptation.^4^ ^,^ ^5^ On the other hand, viruses constrain the host to produce hundreds to thousands of proteins involved in antiviral defense, virus replication, and shedding.^6^ These molecular interactions drive adaptation. This essay discusses the features of the adaptation process of SARS-CoV-2 to humans, its mechanisms, and the virus’s possible evolutionary trajectories.

The process of adaptation of SARS-CoV-2 to humans

The origin and zoonotic route of SARS-CoV-2 to humans remain speculative.^7^ ^,^ ^8^ ^,^ ^9^ ^,^ ^10^ ^,^ ^11^ Several SARS-related coronaviruses (SARSr-CoVs) have been detected in *Rhinolophus* bats from Southern China and have been considered the most likely ancestors of SARS-CoV-2.^11^ Bat-CoV-RaTG13, identified in horseshoe bats (*Rhinolophus affinis*) in Yunnan, China, exhibits so far the highest sequence identity to SARS-CoV-2 at the whole-genome level (96%) and clusters with SARS-CoV-2 in phylogenetic analyses.^12^ However, SARSr-CoVs spike (S) protein diverges in the receptor-binding domain (RBD) of SARS-CoV-2 and shows substantially lower affinity to the human angiotensin-converting enzyme 2 (ACE2) receptor.^13^ Phylogenetic comparative analyses of bat SARSr-CoV and SARS-CoV-2 estimate that they shared a most recent common ancestor approximately 40-50 years ago, indicating that the lineage giving rise to SARS-CoV-2 has been circulating unnoticed in bats for decades.^14^ ^,^ ^15^ ^)^ It is estimated that more than two-thirds of SARS-CoV-2-like zoonotic events would be self-limited, dying out without eliciting a pandemic.^16^ The finding of antibodies to bat coronaviruses in inhabitants of rural areas of China^17^ ^,^ ^18^ before the emergence of SARS-CoV-2 in December 2019 gives support to such estimation.

The search for an intermediate host of SARS-CoV-2 has resulted in frustrating outcomes.^8^ ^,^ ^10^ ^,^ ^19^ ^,^ ^20^ ^,^ ^21^ A possible explanation is that this virus has no intermediate host. Indeed, it is plausible that a progenitor of SARS-CoV-2 had jumped into humans, acquiring its genomic features through adaptation during undetected successive human-to-human transmissions.^8^ ^,^ ^9^ ^,^ ^22^ Such adaptation is not likely to have emerged suddenly but, instead, may have evolved step by step, each one favored by natural selection.^9^ The virus gradually has ‘learned’ how to overcome the respiratory defense barriers. After this process, SARS-CoV-2 was possibly prepared to give rise to full-blown infections and enabled the pandemic to take off to produce a sufficiently large cluster of cases to trigger the surveillance system that detected it.^8^

Moving from bat to human had exposed the virus to an unfamiliar environment requiring numerous adaptive changes, such as receptor-specific adjustments, optimising host factors’ compatibility, and overcoming host antiviral defenses.^23^ This adaptation process relies basically on mutations. The best-characterised mutation of SARS-CoV-2 is D614G, a non-synonymous substitution (nucleotide mutation that alters the amino acid sequence of a protein), resulting in a replacement of aspartic acid (D) with glycine (G) at position 614 of the virus’s spike protein. D614G was first detected in February 2020, and a few months later, over 97% of isolates of SARS-CoV-2 worldwide were found to contain this mutation.^24^ ^,^ ^25^ ^,^ ^26^ Structural models predict that D614G variants present alterations of the S protein conformation so that their binding and fusion to ACE2 receptors are increased.^26^ The D614G mutation is associated with higher viral load and transmissibility of SARS-CoV-2 but not with greater severity of the infection, which is consistent with a selective advantage for the virus.^27^

About 30% of SARS-CoV-2 mutations are synonymous, which leads to modification of the target codon without affecting the associated protein sequence on mRNA translation. Therefore, over time, the virus has adapted its codon usage to that of humans through the accumulation of silent mutations.^28^

Over a year into the pandemic, sequencing analyses show that variants of SARS-CoV-2 are being selected as the virus continues to circulate widely. The predominant drivers of genetic variation within SARS-CoV-2 are single nucleotide polymorphisms caused by polymerase errors, potential host factor-driven RNA modification, and insertion/deletions resulting from the discontinuous nature of viral RNA synthesis.^29^ Based on the analysis of 815,402 high-quality SARS-CoV-2 genome sequences obtained up to March 1, 2021, 8065 single nucleotide variants, 173 deletions, and 49 insertions were identified.^30^

The ongoing evolution of SARS-CoV-2 during the pandemic is primarily characterised by purifying or negative selection, which consists of the selective removal of deleterious alleles. Still, a small set of sites appear to evolve under positive selection.^31^ Positive or directional selection is a modality of natural selection in which advantageous alleles are favored over others, leading to a shift in allele frequency. The receptor-binding domain (RBD) of SARS-CoV-2 spike protein and the region of the nucleocapsid protein associated with nuclear localisation signals are enriched with positively selected amino acid replacements.^31^

Hence, a plausible picture of the process of adaptation of SARS-CoV-2 to humans could be drawn. Adaptation may have started decades ago when the SARS-CoV-2 ancestors in bats might have spilled over to humans on several occasions. During these contacts, the virus possibly found opportunities to evolve gradually to improve its access to the novel hosts’ cells, take advantage of their resources, and overcome their defense mechanisms. Eventually, virus variants had emerged capable of replicating and shedding in the novel host and being transmitted to other humans. The adaptation process of SARS-CoV-2 is still progressing, and its natural trend is the progressive reduction of the mutual aggressions between virus and host.

Mechanisms of adaptation of SARS-CoV-2

Months after its emergence, SARS-CoV-2 had infected hundreds of millions of people. That high transmissibility denotes the ability to replicate and shed in humans, which means adaptation. That implies that SARS-CoV-2 has developed strategies to overcome the human respiratory defense barriers. These include the mucus and mucociliary clearance, surfactant proteins, the respiratory tract microbiota, defensins and lactoferrin, secretory IgA antibodies, interferons, innate immunity, and adaptive immunity. How the SARS-CoV-2 evades the first five barriers is essentially unknown. However, the mechanisms used by the virus to overcome the interferon barrier and the innate and adaptive immunity are well established. They can be divided into two categories: mechanisms of escape and utilisation of host resources. The strategies of escape of SARS-CoV-2 from the human defense barriers include impairment of interferon production, hiding of immunogenic motifs, avoidance of viral RNA detection, manipulation of cell autophagy, triggering host cell death, induction of lymphocyte depletion, induction of lymphocyte exhaustion, and virus mutation. While the strategies of host resource usage encompass inhibition of the ubiquitin-proteasome system, hijacking of mitochondria functions, and usage of enhancing antibodies (see Table I).

**TABLE I:** Mechanisms of adaptation of severe acute respiratory syndrome coronavirus 2 (SARS-CoV-2) to humans

Escape from defense barriers	Targets
Impairment of interferon production	Epithelial cells / Innate immunity cells
Hiding of immunogenic motifs	Epithelial cells
Avoidance of viral RNA detection	Epithelial cells
Manipulation of cell autophagy	Epithelial cells / Innate immunity cells
Triggering of host cell death	Epithelial cells / Innate immunity cells
Induction of lymphocyte depletion	Adaptive immunity cells
Induction of lymphocyte exhaustion	Adaptive immunity cells
Virus mutation	Adaptive immunity
Host resource usage	Targets
Inhibition of the ubiquitin-proteasome system	Epithelial cells
Hijacking of mitochondria functions	Epithelial cells / Innate immunity cells
Usage of enhancing antibodies	Adaptive immunity

*Impairment of interferon production* - Types I and III Interferons (IFNs) are cytokines endowed with potent antiviral and immunomodulatory effects produced by the innate immune cells and respiratory epithelial cells.^32^ ^,^ ^33^ They act by inhibiting virus entry, modulating membrane lipids to prevent viral release, inducing apoptosis of infected cells, and regulating transcriptional and posttranscriptional mechanisms, as well as posttranslational events.^34^ ^,^ ^35^ The immunomodulatory effects of IFNs rely on their ability to interfere with virtually all immune cells’ functions.^36^ They upregulate many pattern-recognition receptors,^37^ ^,^ ^38^ drive and amplify pro- and anti-inflammatory responses,^39^ ^,^ ^40^ and enhance immune responses by activating and recruiting immune cells.^41^ ^,^ ^42^ ^,^ ^43^ The reduced or delayed production of types I and III IFNs by the respiratory epithelium and innate immune cells in both SARS-CoV-1^44^ and SARS-CoV-2 infections^45^ hinders virus clearance and is probably associated with developing the cytokine release syndrome, a major cause of mortality of coronavirus disease-19 (COVID-19) and severe acute respiratory syndrome (SARS).^46^ ^,^ ^47^

It has been found that SARS-CoV-2 inhibits the expression of types I and III IFNs *in vitro*, in cell cultures,^45^ *ex vivo*, in human lung tissue explants,^47^ and *in vivo*, in individuals with COVID-19.^48^ IFN inhibition is higher in those with severe disease associated with persistent blood virus load and exacerbated inflammatory response.^48^ ^,^ ^49^ SARS-CoV-2 uses different proteins to inhibit IFN production. They include NSP (non-structural protein) 13 (helicase), NSP14 (exonuclease), NSP15 (endoribonuclease), and accessory protein ORF (open reading frame) 3b.^50^ ^,^ ^51^ However, ORF-6 displays the strongest antagonistic effect on both IFN production and signaling.^51^ ^,^ ^52^ ^,^ ^53^ The ability to impair IFN production depends on the particular SARS-CoV-2 strain. A variant found in 37 countries worldwide containing a deletion in the Nsp1-coding region (Δ500-532) induced much lower serum IFN-β levels in infected individuals than viruses not showing such deletion.^54^

*Hiding of immunogenic motifs* - During the ~10 minutes, a SARS-CoV-2 particle takes to enter the host cell and ~10 hours to complete the intracellular replication to give rise to ~10^3^ virions,^55^ it appears as if a molecular war is occurring. The ‘combatants’ - the virus and host cell - use sophisticated attacks and counterattack strategies to reach their ultimate survival goal. The confrontation starts at the point of the contenders’ encounter - the cell membrane - where the virus’s spike (S) protein meets angiotensin-converting enzyme 2 (ACE2) molecules, the receptors for its entry into the cell.

To enter host cells, SARS-CoVs use the two subunits of S protein: the S1 subunit, where the RBD is located, and the S2 subunit, which allows the virus to fusion to the cell membrane after priming by cellular protease.^56^ SARS-CoVs’ RBD continually switches between a standing-up position at the S1 subunit, which enables receptor binding, and a lying-down position, which does not bind to the host receptor.^57^ ^,^ ^58^ This insignificant molecular detail has significant consequences for the infectivity, evasiveness, spreading, pathogenicity, and control of SARS-CoVs.

The distinct RBD positions in SARS-CoV-2 and SARS-CoV-1 explain some differences between these viruses. While in SARS-CoV-1, the RBD is mainly in the standing-up state,^57^ ^,^ ^59^ in SARS-CoV-2 it is mainly in a lying-down state.^60^ ^,^ ^61^ This feature provides an advantage for the last virus: it hides the most immunogenic region of S protein from the immune system’s cells. Hence, it hinders the synthesis of neutralising antibodies against the virus and makes it difficult to access these antibodies to the target,^62^ which poses a challenge to both vaccination and plasma therapy. Thus, the RBD position acts as a mechanism of evasion of SARS-CoV-2 from the immune response, contributing to increasing the viral load and the virus’s pathogenicity.^58^

*Avoidance of viral RNA detection* - Depending on the protease availability and the cell type, SARS-CoV-2 utilises two different routes to infect the host cell: the cell membrane pathway and the endosomal pathway. When the cell membrane is rich in transmembrane protease serine 2 (TMPRSS2), as occurs with respiratory epithelial cells, the membrane pathway is the usual route. TMPRSS2 activates the S protein and promotes the virus fusion to the cell membrane receptor, leading to the viral RNA’s release into the cytosol.^56^ In the cell’s interior, the virus’s components are synthesised in the rough endoplasmic reticulum (ER), followed by their assembly in the ER-Golgi intermediate compartment. Finally, virus copies are released from the cell.

The endosomal pathway is used when proteases are not sufficiently available at cell membranes, as frequently occurs with cells outside the respiratory tract. In this case, the entire virion is endocytosed. Within the endosome, the low pH activates cathepsin L that cleaves S protein and triggers the fusion pathway, leading to the viral RNA’s release into the cytoplasm, where it follows the same order of events used in the plasma membrane pathway.^63^ ^,^ ^64^

During the hours the virus takes to complete its replication cycle inside the host cell, several maneuvers of the ‘molecular war’ between the virus and the cell occur. The main protagonist of this ‘war’ is the viral RNA released into the cell cytosol. For the virus, it is the warranty for the production of new viral particles, and for the cell, it represents a dangerous intruder capable of interfering with vital cell functions. Host cells need to recognise and destroy the viral RNA to avoid virus replication and their destruction by the virus. Conversely, the virus needs to circumvent its RNA recognition and destruction to induce a productive infection.^65^

Cells display several sensors capable of detecting viral RNA either in endosomes (e.g., Toll-like receptors or TLRs) or spread in the cytosol (e.g., the retinoic acid-inducible gene I (RIG-I)-like receptors or RLRs). These receptors’ sensing of viral RNA functions as alarm signals and gives rise to interferon type I (IFN-I) production, which has two consequences. First, IFN-I can bind its cellular receptor to induce a signaling cascade that culminates with the expression of hundreds of IFN-stimulated genes. These gene products generate an antiviral state in the cell that hinders virus development and spread. Second, IFN-I promotes the synthesis of cytokines and chemokines that attract and activate cells of the innate immune response endowed with antivirus activity.^66^ ^,^ ^67^

So crucial is the integrity of the viral nucleoprotein that SAS-CoVs exhibit different strategies to protect their RNA molecules to avoid detection by cell RNA sensors and destruction by IFNs.^65^ ^,^ ^68^ Viral RNA can be either enclosed into cell double-membrane vesicles^69^ or mimicked into cellular mRNA by adding a cell cap structure to its molecule.^65^ ^,^ ^70^ ^,^ ^71^ Alternatively, the virus uses its endoribonuclease activity to destroy the cell RNA sensors.^72^ ^,^ ^73^ ^,^ ^74^

*Manipulation of cell autophagy* - SARS-CoVs employ different strategies to prevent their destruction by interfering with some critical host cell functions. Manipulation of cell autophagy is one of them. Autophagy is a process used by cells to eliminate harmful, superfluous, or defective cell components and generate new macromolecules or bioenergetics reactions. As part of innate immune defenses, autophagy targets viruses and viral components for lysosomal degradation and exposes pathogen-associated molecular patterns to facilitate recognition.^75^ ^,^ ^76^ ^,^ ^77^ Therefore, viruses evolved sophisticated strategies to antagonise autophagy and even exploit it to promote their replication.^75^ ^,^ ^77^_^,78)^_

SARS-CoV-2 uses several proteins to manipulate cell autophagy.^77^_^,78,^_ ^79^ ^,^ ^80^ The two more important are ORF3a, which prevents the fusion between autophagosomes and lysosomes,^77^ ^,^ ^79^ ^,^ ^80^ and ORF7a, which reduces the acidity of lysosomes.^77^

*Triggering of host cell death* - Host cell death is a common outcome of virus infection. In some cases, it curbs virus replication; in others, cell death promotes virus dissemination and contributes to tissue injury and disease. Viruses can elicit three forms of cell death: apoptosis, necroptosis, and pyroptosis. Recent works have introduced the term PANoptosis to encompass the three frequently associated mechanisms.^81^ ^,^ ^82^

Apoptosis is the most extensively studied cell death mechanism in virus infections. Cells undergoing apoptosis induced by different caspases exhibit characteristic features, including condensation of chromatin, fragmentation of DNA, exposure of phosphatidylserine on the plasma membrane, and formation of apoptotic bodies with contents of the cell enclosed within a membrane.^83^ Usually, apoptosis is an antiviral pathway activated by host cells to limit virus replication.

Depending on the virus, proapoptotic signaling can be initiated at any stage of infection: at the viral attachment to the host cell; during viral entry; by viral nucleic acids present in the cytosol, mitochondria, or nuclear compartments; and by viral gene expression.^83^ While the induction of apoptosis may favor viral dissemination at the late stages of infection, viruses inhibit it at the early steps of the infectious cycle, thereby avoiding premature cell death and allowing virus replication. For that, viruses have developed a battery of Bcl-2 homologs by which they mimic the major antiapoptotic system of host cells. However, they can employ several other mechanisms for inhibiting cell apoptosis.^84^ Conversely, viruses may take advantage of stimulating apoptosis through the activation of caspases, either to kill uninfected cells of the immune system or to induce the breakdown of infected cells, in that way either favoring viral dissemination.^85^ ^,^ ^86^

Coronaviruses can either induce cell apoptosis^87^ ^,^ ^88^ ^,^ ^89^ or inhibit it,^90^ ^,^ ^91^ depending on the viral load and the subcellular localisation of viral RNA.^92^ The accessory protein ORF3a encoded both by SARS-CoV-1,^93^ ^,^ ^94^ and SARS-CoV-2^95^ ^,^ ^96^ can efficiently induce apoptosis in cell cultures. SARS-CoV-2 ORF3a induces the cleavage/activation of caspase-8, whereas Bcl-2 expression levels were not affected, a hallmark of the extrinsic apoptotic pathway.^95^

Apoptosis inhibition can trigger another host defense response, antiviral necroptosis.^86^ Necroptosis, or programmed necrosis, shares the same TNF receptor with apoptosis but, different from the latter in which the caspase pathway is activated, it is inhibited.^97^ Necroptosis consists of swelling of organelles and rupture of the plasma membrane to release intracellular contents and requires the activity of receptor-interacting protein kinases 1 and 3 (RIPK1, RIPK3).^83^ ^,^ ^98^ ^,^ ^99^ Furthermore, unlike apoptosis, which leads to a noninflammatory effect, necroptosis releases cellular factors that promote the recruitment and activation of immune cells.^100^ ^,^ ^101^ Depending on the virus and its interactions with the cell, necroptosis can either suppress or promote viral growth. Accordingly, in certain circumstances, the virus can facilitate it, and in others, inhibit it.^86^ ^,^ ^102^ Little is known about the occurrence of necroptosis during SARS-CoV infections. However, the lung inflammatory syndrome associated with SARS-CoV-1^86^ and SARS-CoV-2^103^ is probably triggered by necroptosis.

The third form of cell death as a reaction to virus infection is pyroptosis. Pyroptosis exhibits features of both apoptosis and necroptosis and is observed mainly in phagocytes. Cells undergoing pyroptosis expose phosphatidylserine in the plasma membrane and have condensed chromatin as apoptotic cells. However, akin to necroptotic cells, they display leak cytoplasmic contents due to damage to the plasma membrane. Caspases, specifically caspase-1, are central players in the induction of pyroptosis since they dimerize within large cytoplasmic complexes - the inflammasomes - that initiate the process of cell death.^83^ ^,^ ^104^ The NOD-, LRR-, and pyrin domain-containing 3 (NLRP3) inflammasome are the most extensively studied among the inflammasomes. It plays a crucial role in both inflammation and antiviral responses. It is activated by sensing viral components and cytosolic danger signals, such as mitochondria injury, protein aggregates, and aberrant ion concentrations, all of which can be produced by viral infections.^105^

The activation of the NRLP3 inflammasome requires two steps. The first, or priming step, is triggered by the binding of viral components to pattern recognition receptors (or TNF or IFN to their cell receptors), leading to the activation of the NF-kB pathway, followed by the expression of NLRP3, pro-IL-1beta, and pro-IL-18. The second step, or activation step, is triggered by the sensing of viral nucleoproteins and cellular byproducts of the infection by cytosolic sensors. This step results in the assembly and oligomerisation of the inflammasome complex and auto-cleavage of pro-caspase-1. Next, caspases 1, 4, and 5 are activated and mediate the proteolytic processing of pro-IL-1beta, pro-IL-18, and propyroptotic factor gasdermin-D (GSDMD). GSDMD forms pores in the plasma membrane of infected cells, facilitating the release of the proinflammatory cytokines IL-1beta and IL-18 and hence completing the process of pyroptosis.^105^ ^,^ ^106^ ^,^ ^107^

Both SARS-CoV-1^108^ ^,^ ^109^ ^,^ ^110^ and SARS-CoV-2^111^ encode viroporins, which activate the NLRP3 inflammasome and induce cell death by pyroptosis.^111^ ^,^ ^112^ This activation is detrimental for the viruses since it inhibits viral replication and promotes viral clearance. SARS-CoV-2 developed a way to overcome it. Its non-structural proteins (NSPs) NSP1 and NSP13 can significantly reduce NLRP3-inflammasome-induced caspase-1 activity and IL-1beta secretion.^113^

*Induction of lymphocyte depletion* - SARS-CoV-2 can cause both quantitative^114^ ^,^ ^115^ ^,^ ^116^ and functional^117^ ^,^ ^118^ ^,^ ^119^ alterations of lymphocytes. Lymphocyte depletion is detected in over 70% of hospitalised patients of COVID-19 and is more accentuated in severe than in mild infection. It encompasses all lymphocyte subsets, such as CD4+ and CD8+ T cells, B cells, and natural killer (NK) cells.^119^ Furthermore, lymphocyte depletion has been demonstrated in infected individuals’ blood, spleen, and lymph nodes.^120^ The reasons for this dramatic destruction of lymphocytes in SARS-CoV-2 infection is a matter of discussion.

Lymphocytes express ACE2^121^ ^,^ ^122^ and CD147 molecules,^123^ ^,^ ^124^ which act as receptors for SARS-CoV-2. The finding that lymphocytes can be infected either *in vitro* or *in vivo* and can sustain virus replication^125^ points to a possible direct effect of SARS-CoV-2 on cell destruction. However, the fact that lymphocytes containing virus nucleoprotein are rarely detected in infected individuals’ tissues^122^ indicates that the direct effect of SARS-CoV-2 is possibly not an effective mechanism of lymphocyte destruction.

The temporal association between lymphocyte depletion and the heightened levels of proinflammatory cytokines, including TNF, IL-6, IL-1beta, IL-12, IL-18, IL-33, and IFN-I, points to the possibility of a cause-effect relationship.^115^ ^,^ ^116^ ^,^ ^119^ ^,^ ^120^ ^,^ ^126^ In fact, it has been found that pro-inflammatory cytokines can inhibit lymphopoiesis^127^ ^,^ ^128^ ^,^ ^129^ and exhibit antiproliferative and proapoptotic effects on lymphocytes,^130^ ^,^ ^131^ ^,^ ^132^ ^,^ ^133^ ^,^ ^134^ particularly CD8+ T cells,^131^ ^,^ ^132^ the most depleted population in SARS-CoV-2 infection. Other possible factors involved in lymphocyte depletion are increased glucocorticoid,^135^ ^,^ ^136^ and lactic acid concentrations^137^ that occur during infection.

*Induction of lymphocyte exhaustion* - The second mechanism that has been suggested to explain SARS-CoV-2 evasion of the immune response is lymphocyte exhaustion. Lymphocyte exhaustion is a dysfunction that arises when the immune cells are exposed to high antigenic loads during infections, transplantation, or cancer.^138^ ^,^ ^139^ ^,^ ^140^ A poor effector function characterises it with the sustained expression of inhibitory receptors and a transcriptional and epigenetic state distinct from that presented by functional effector and memory lymphocytes.^139^ ^,^ ^141^ During the process of exhaustion, the loss of function of lymphocytes occurs hierarchically. It starts with the impairment of IL-2 production and proliferative capacity, followed by the decline of TNF, IFN-γ, and beta-chemokine synthesis, reduced responsiveness to inflammatory cytokines, and the complete loss of functionality of the cell and eventually its deletion.^139^ ^,^ ^142^ These stepwise dysfunctions are accompanied by a progressive increase in the amount and diversity of expression of inhibitory receptors, including PD-1 (CD279), CTLA-4 (CD152), LAG-3 (CD223), Tim-3, 2B4 (CD244), CD160, and TIGIT.^139^ ^,^ ^140^ ^,^ ^142^ ^,^ ^143^ The exhaustion process includes critical metabolic changes in lymphocytes characterised by shifting from glycolytic toward oxidative phosphorylation pathways and the disruption of mitochondrial organisation and function, which impact their long-term survival and contribute to the failure to establishing immune memory.^143^ ^,^ ^144^ Lymphocyte exhaustion can affect T and B lymphocytes and NK cells. It is driven by different factors that include persistent or intense antigen exposure, deficient CD4 lymphocyte help, low dendritic cell activation, the exaggerated effect of regulatory T lymphocytes, reduced co-stimulation, increased IL-10 and TGF-β, diminished IL-2, IL-7, IL-21 signals, persistent IFN-I and TNF signals, and exaggerated action of NK cells.^143^ ^,^ ^145^ ^,^ ^146^

Several authors have interpreted the up-regulation of inhibitory receptors by CD8+, CD4+, and B lymphocytes and NK cells in SARS-CoV-2 infection to indicate cell exhaustion.^115^ ^,^ ^117^ ^,^ ^147^ ^-^ ^153^ However, this conclusion should be interpreted with caution since up-regulation of inhibitory receptors is a bona fide marker of lymphocyte activation and does not necessarily indicate the lymphocyte dysfunction characteristic of exhaustion.^154^ Nonetheless, lymphocyte functional impairment has been recently described in SARS-CoV-2-infected individuals with severe disease,^155^ as shown in other viral respiratory infections.^156^ ^,^ ^157^ ^,^ ^158^ ^,^ ^159^

*Virus mutation and escape from immunity* - Mutation of its antigenic repertoire is another mechanism of SARS-CoV-2 evasion from the host immune response. Mutations are inevitable consequences of being a virus. Viruses depend on mutations to fuel their variability and thus enable adaptability and evolutionary changes. Based on their effects on fitness, mutations can be divided into three broad categories: the ‘good’ or advantageous that increase fitness, the ‘bad’ or deleterious that decrease it, and the ‘indifferent’ or neutral that are not affected by selection because their effects are too small.^160^ Most mutations are neutral or deleterious, and only a few are beneficial for viruses.^160^ ^,^ ^161^ The beneficial mutations include those that allow viruses to evade immune responses.^162^ ^,^ ^163^ ^,^ ^164^ Hence, viruses showing high mutation rates tend to evade immunity more efficiently.^165^

SARS-CoVs are single-stranded RNA viruses that are especially apt to take advantage of mutations to adapt to new hosts and environments. RNA viruses mutate faster than DNA viruses, and single-stranded viruses mutate faster than double-stranded viruses.^165^ ^,^ ^166^ High mutation rates can be advantageous or detrimental for the virus. On the one hand, it may enhance virulence and evolvability. But, on the other hand, it may cause viral extinction due to the accumulation of mutations during copying cycles.^161^ ^,^ ^167^ While RNA viruses have long been considered unable to correct errors during replication, SARS-CoVs are important exceptions since they encode an exoribonuclease that prevents or removes misincorporated nucleotides during mutagenesis.^168^ Mutations detected in SARS-CoV-2 spike surface glycoproteins may change their antigenicity, increase viral load and transmission, and facilitate virus evasion.^4^ ^,^ ^169^ ^,^ ^170^ ^,^ ^171^ Lineages of heavily mutated viruses containing such detrimental mutations represent public health hazards and are termed ‘variants of concern’. Table II displays the main features of SARS-CoV-2 variants of concern.

**TABLE II:** Main features of severe acute respiratory syndrome coronavirus 2 (SARS-CoV-2) variants of concern and of interest

Pango lineages	WHO label	First documented	Features
B.1.1.7	Alpha	UK	Higher transmissibility (70%) and lethality (60%) Moderate reduction of neutralisation efficiency
B.1.351	Beta	South Africa	Higher transmissibility (20-113%) Significant reduction of neutralisation efficiency
B.1.617.1 B.1.617.2	Kappa Delta	India	Reduction of neutralisation efficiency
B.1.1.284 (P.1)	Gamma	Brazil	Higher transmissibility (160%) and lethality (80%) Reduction of neutralisation efficiency
B.1.427 B.1.429	Epsilon	USA	Higher transmissibility (20%) Moderate reduction of neutralisation efficiency

Adapted from Soh et al.^172^

As highly deleterious mutations are rapidly purged, most observed mutations are either neutral or mildly deleterious.^173^ Mutations contributing to the virus fitness advantage tend to be a minority compared with low- or no-effect neutral amino acid changes.^174^ Such mutations may alter various aspects of virus biology, as pathogenicity, infectivity, transmissibility, and antigenicity.

It is well established that SARS-CoV-2 variants may escape immunity.^175^ ^,^ ^176^ ^,^ ^177^ ^,^ ^178^ However, to which extent can active (post-infection or post-vaccination) or passive (convalescent plasma or monoclonal antibodies) immunity drive mutations by inducing viral antigenic changes or selecting virus variants? Phylogenetics, bioinformatics, and mathematical processing have estimated that during SARS-CoV-2 infection, over 95% of virus samples show within-host mutations.^179^ Discrete genetic variants appear de novo, and, over time, some variants disappear due to purifying selection against nonsense mutations, while others persist and are transmitted.^179^ ^,^ ^180^ It has been found low within-host diversity when viral loads are high.^180^ If one considers the immune system’s role, this apparent paradox can be solved. Indeed, during early infection, the viral load is high, and the virus diversity is low. However, as long as the immune system is activated during the infection, it may control the viral load^181^ ^,^ ^182^ and exert selective pressure on the virus population, thus increasing its diversity.^183^ ^,^ ^184^ ^,^ ^185^ ^,^ ^186^ Mutations likely arise when the SARS-CoV-2 is put under selective pressure by antibodies that limit but do not eliminate viral replication. Under these conditions, the virus might find a way to escape this pressure and restore its reproduction ability more efficiently.^187^ Immune-induced SARS-CoV-2 mutations have been described in association with convalescent plasma *in vitro* ^188^ and *in vivo* ^189^ ^,^ ^190^ ^,^ ^191^ ^,^ ^192^ as well as with the anti-COVID-19 vaccine.^193^

*Inhibition of the ubiquitin-proteasome system* - The ubiquitin-proteasome system (UPS) plays a central role in a wide range of fundamental cellular functions by ensuring protein quality control and maintaining a critical level of regulatory proteins.^194^ It modulates many fundamental cellular processes, including cell cycle, signal transduction, transcriptional regulation, antigen processing, and apoptosis.^195^ The UPS plays opposite roles in viral infection. On the one hand, it benefits the virus by controlling viral proteins’ stability. On the other, the UPS mediates viral protein degradation and the production of antiviral molecules such as interferons.^194^ ^,^ ^196^

Viruses use two strategies for manipulating the UPS for their benefit. First, they target key host immune molecules (e.g., type I interferon and class I MHC) for proteasomal degradation. Second, they prevent the destruction of inhibitors of transcription factors critical for the immune response as NFκB.^196^

Both SARS-CoV-1^197^ ^,^ ^198^ and SARS-CoV-2^199^ encode a papain-like protease endowed with the capacity to inhibit both ubiquitin and ubiquitin-like (ISG15) activities and, hence, counteract the posttranslational modification of signaling molecules that activate an antiviral response.

*Hijacking of mitochondria functions* - Mitochondria are essential cell organelles involved in various functions, from energy production and fatty acid oxidation to cell cycle regulation and death. They carry out critical signaling processes in the synthesis of anti-infectious molecules such as reactive oxygen species, interferons, and proinflammatory cytokines.^200^ Therefore, several infectious agents developed strategies to disrupt mitochondria functions to preserve themselves.^201^ ^,^ ^202^ As obligate intracellular pathogens, viruses primarily depend on mitochondria functions and are vulnerable to their anti-infectious effects. Hence, they have developed various mechanisms aiming to optimise or inhibit mitochondria’s functions for their benefit. They include regulation of mitochondrial membrane potential and calcium homeostasis, control of oxidative stress, hijacking of mitochondrial proteins, alteration of mitochondria distribution, depletion of mitochondrial DNA, mimicking of mitochondrial proteins, triggering and manipulation of mitophagy, and regulation of apoptosis.^202^ ^,^ ^203^

Both *in vitro* cell infections and computational modeling show that SARS-CoV-2 can sequester mitochondrial functions. By comparing hundreds of SARS-CoV-2 genomes to the human transcriptome, it was found that the viral RNA is enriched in the host mitochondrial matrix and nucleolus, where the virus can hijack the existing cell machinery for its replication and escape from detection by cytosol RNA sensors.^204^ The association of SARS-CoV-2 with the mitochondrial matrix and nuclear compartments is much higher than other coronaviruses and could play a role in its greater infectivity.^204^ ^,^ ^205^ Different ORF products of SARS-CoV-2 can induce mitochondrial DNA (mtDNA) release in infected cells’ cytoplasm and activate mtDNA-induced inflammasome, which interferes with both the innate and the adaptive immunity.^206^

*Usage of enhancing antibodies* - The antibody-dependent enhancement (ADE) phenomenon facilitates viral entry into host cells and enhances the infection’s breadth.^207^ ADE occurs in coronavirus infections, including MERS-CoV,^208^ SARS-CoV-1,^209^ ^,^ ^210^ and SARS-CoV-2 infections.^211^ ^,^ ^212^ ^,^ ^213^ ^,^ ^214^ ^,^ ^215^ It ensues when antibodies binding to the virion fail to efficiently neutralise the virus, either because they bind to viral epitopes other than those involved in cell attachment and entry or due to the presence of sub-neutralising concentrations of antibodies.^215^ The complex virus-antibody (IgG, IgM, or IgA), with or without complement, is internalised into those cells expressing Fc or C receptors, such as lymphocytes, monocytes, macrophages, dendritic cells, granulocytes, and endothelial cells. Viruses’ ability to gain access to ACE2-negative cells significantly augments viral loads, broadens their capability to exploit new resources, and causes functional alteration of infected cells, which ultimately exacerbates viral pathogenesis and contributes to disease severity.^216^ ^,^ ^217^

The ability of SARS-CoVs to invade cells that express Fc or C receptors has been demonstrated both *in vitro* and *in vivo*.^125^ ^,^ ^210^ ^,^ ^218^ ^,^ ^219^ Although replication does not always occur at a sustained high level, the virus’s simple presence in the host cell’s interior can alter its function and viability.^220^ ^,^ ^221^ ^,^ ^222^ ^,^ ^223^ ADE possibly plays a role in SARS-CoV infection pathological mechanisms, such as lymphopenia, cytokine storm, and lung involvement.^211^ ^,^ ^221^ ^,^ ^224^ The demonstration of the second peak of throat and nasal virus load after the 9th day post-infection, when antibodies to SARS-CoV-2 start to be detected^224^ and the association between seroconversion and worsening of the clinical condition^225^ ^,^ ^226^ are highly suggestive of the involvement of the ADE phenomenon.

The possible evolutionary trajectories of SARS-CoV-2

*Different evolutionary trajectories of coronaviruses* - Human coronaviruses are zoonotic pathogens that cause either mild endemic and seasonal common cold (HCoV-229E, HCoV-OC43, HCoV-NL63, and HCoV-HKU1) or severe epidemic disease (SARS-CoV-1, MERS-CoV, and SARS-CoV-2). Several factors explain these clinical and epidemiological differences. A critical aspect is how long the viruses had emerged from their animal hosts. Molecular clock analyses estimate that the endemic coronaviruses are much older than the epidemic ones. HCoV-HKU1 probably emerged in the early 1950s, while HCoV-OC43 diverged roughly 120 years ago, HCoV-229E about 200 years, and HCoV-NL63 is predicted to have existed for 560 to 820 years.^227^ ^,^ ^228^ ^,^ ^229^ Within the epidemic coronaviruses, SARS-CoV-1 had probably diverged from 4 to 17 years before the 2002 epidemic, MERS-CoV about 2006,^227^ and SARS-CoV-2 between mid-October and mid-November 2019.^16^ These different evolutionary times help explain why the ‘old’ common cold coronaviruses are well adapted while the ‘young’ SARS-CoV-2 is in its destructive trajectory, despite vaccination.

The evolutionary trajectories also widely differ within the same group of ‘young’ coronavirus. MERS-CoV, first isolated in Saudi Arabia in 2012, had infected, seven years later, 2574 individuals from 27 countries (75% from Saudi Arabia) and caused 858 deaths (case fatality rate of 33%). The transmission by dromedary camels is kept locally active, and the infection follows an endemic pattern.^230^ ^,^ ^231^ The trajectory of SARS-CoV-1 was quite unexpected. The major outbreak started in November 2002 as a rapid epidemic in China, spread through 29 regions worldwide, and resulted in more than 8000 cases and 774 deaths.^232^ The epidemic was effectively controlled under vigorous quarantine measures, and no new case was reported after July 2003. Six months after its disappearance, SARS-CoV-1 re-emerged as four sporadic cases in Guangdong Province, China, causing no fatality or secondary transmission.^233^ The prompt action of the Chinese authorities in the isolation of suspect cases and in instigating contact tracing and quarantine measures served to contain the virus effectively.^234^ However, health authorities were helped by two biological characteristics of SARS-CoV-1 infection: its low transmissibility and the absence of transmission by asymptomatic individuals, which enabled effective diagnosis and isolation.^234^

Thus, three related coronaviruses followed three completely different evolutionary trajectories: MERS-CoV gave rise to a restricted and prolonged endemic, SARS-CoV-1 to a rapidly eliminated epidemic, and SARS-CoV-2 to a hardly contained pandemic.

*Possible future scenarios for SARS-CoV-2 and COVID-19* - What is to be expected about the future of SARS-CoV-2 and COVID-19? Will the virus become more virulent and make the pandemic worse? Or will it be eliminated, as has happened with SARS-CoV-1? Or, will COVID-19 become an endemic disease with seasonal outbreaks, as occurs with common cold coronaviruses? Or, finally, will SARS-CoV-2 become part of the respiratory virome and coevolve with humans?

Virulence, understood as the severity or harmfulness of a pathogen,^235^ evolves with the virus. However, notwithstanding the extensive research of evolutionary biologists, the rules commanding the evolution of virulence have not been firmly established. Therefore, the ability to make robust predictions is minimal. A basic tenet as the evolution of virulence following the jump of a virus to a new host species continues to be contentious.^235^ It is known that the severity of disease caused by SARS-CoV-2 is bound to decrease with increasing population immunity.^236^ However, that is the expression of the efficiency of immunity, and little informs about the virus virulence.

The virulence of a pathogen is usually inferred by the mortality it causes in the host.^237^ The enormous casualties associated with COVID-19 are frequently interpreted as indicating SARS-CoV-2 high mortality rates. That is misleading. The baseline estimates of SARS-CoV-2 infection-fatality ratio for those below 70 years old is 2.9% (2.4%-3.5%),^238^ much lower than that of SARS-CoV-1, MERS-CoV, or influenza. Therefore, SARS-CoV-2 should be considered a low-virulence pathogen. The increased virulence of some virus variants is regarded as a transitory phenomenon due to host immunity constraints.^239^ Since, at present, there is a lack of compelling evidence of an overall increase of SARS-CoV-2 virulence,^240^ ^,^ ^241^ ^,^ ^242^ ^,^ ^243^ there is no plausible reason to expect a future worsening of the COVID-19 pandemic.

The second possible scenario for SARS-CoV-2 is to be eliminated, as has happened with the SARS-CoV-1. Disease elimination is the absence of sustained endemic transmission in a country or other geographical region, whereas eradication is the global reduction of infection to zero cases. The only infectious disease that has been so far eradicated was smallpox. The fact that it had no intermediate host or reservoir was decisive. Both eradication and elimination require favorable epidemiological and immunological conditions and a plethora of financial and organisational resources. However, the more restricted area involved in elimination makes it a more achievable goal.

The severe acute respiratory syndrome (SARS) epidemic of 2003 caused by SARS-CoV-1 was rapidly contained through syndromic surveillance, prompt isolation of patients, strict enforcement of quarantine of all contacts, and in some areas, top-down enforcement of community quarantine. By interrupting all human-to-human transmission, SARS was effectively eliminated.^244^ COVID-19 differs from SARS in terms of the infectious period, clinical severity, transmissibility, and extent of community spread.^234^ ^,^ ^244^ These profound differences impede the application to SARS-CoV-2, the same strategies successfully used to eliminate SARS-CoV-1.

The fourth scenario is that COVID-19 will become an endemic disease capable of causing seasonal outbreaks like the common cold coronaviruses. This adaptive trajectory has been previously followed by the 2009 influenza pandemic H1N1 virus and the four known seasonal coronaviruses,^245^ ^,^ ^246^ and, according to mathematical modeling studies, the same will soon occur with SARS-CoV-2.^247^ ^,^ ^248^ A model predicts that the initial SARS-CoV-2 outbreak of severe, high-shedding infections will be followed by an endemic state characterised by mild, low-shedding infections like the seasonal coronaviruses.^248^ Experimental evidence shows that SARS-CoV-2 is environmentally sensitive and, therefore, this characteristic could affect its transmission.^249^

Finally, the last possible scenario is that SARS-CoV-2 becomes part of the respiratory virome and eventually coevolve with humans. The prevailing paradigm in immunology assumes that an effective immune response acts on the infectious agent to avoid the infection’s success. A novel proposal has challenged this concept: immunity aims to facilitate the permanence of infectious agents in the organism with minimal harm and provide mutual adaptation.^182^ That means transforming pathogens into microbiota and, thus, promoting symbiosis (“living together”) and coadaptation. Through this state of coadaptation, conditions are created for promoting coevolution.^2^ ^,^ ^250^ ^,^ ^251^ Therefore, the immune system acts to integrate microbes into the animal-cell community. In the case of SARS-CoV-2, to transform it into virome.

At its arrival to the respiratory virome, SARS-CoV-2 will find all four common-cold coronaviruses still there, as inhabitants of both the upper and lower respiratory tract.^252^ ^,^ ^253^ ^,^ ^254^ ^,^ ^255^ ^,^ ^256^ However, even before that, SARS-CoV-2 was suffering the effects of the coronaviruses from the respiratory virome. These viruses contribute to forming the long-lasting memory B-cell pool that contributes to the early production of anti-SARS-CoV-2 neutralising antibodies.^257^ ^,^ ^258^ In short, they were performing their coevolutionary role.

*In conclusion* - SARS-CoV-2 displays several mechanisms of adaptation that enable it to escape the human defense barriers and take advantage of host cell resources for its profit. Like other RNA viruses, it shows a high proneness to mutate and, therefore, to adapt to new hosts and environments. Since zoonotic pathogens are often poorly adapted following a host shift,^240^ they are expected to cause severe damage to humans during the initial phase of adaptation to the novel host, as have occurred with avian flu, Ebola, and Zika viruses.^235^ Evolutionary models indicate that SARS-CoV-2 suffers a robust natural selection as it spreads in the human host. Its high transmission rate, characteristic of this adaptation phase, tends to speed up virus fitness through mutations.^240^ Although several variants of the virus have been detected worldwide, some associated with increased viral loads and transmissibility, there is so far no compelling evidence of an overall increase of SARS-CoV-2 virulence capable of leading to a worsening of the pandemic.^240^ ^,^ ^241^ ^,^ ^242^ ^,^ ^243^ ^,^ ^259^ ^,^ ^260^

The most plausible scenario for SARS-CoV-2 evolution is to become part of the respiratory tract virome and occasionally give rise to seasonal outbreaks of common cold, as happens with the four other coronaviruses.

## References

[1] Lederberg J (1997). Infectious disease as an evolutionary paradigm. Emerg Infect Dis.

[2] Tosta CE (2001). Coevolutionary networks a novel approach to understanding the relationships of humans with the infectious agents. Mem Inst Oswaldo Cruz.

[3] Eberl G (2010). A new vision of immunity homeostasis of the superorganism. Mucosal Immunol.

[4] Yao H, Lu X, Chen Q, Xu K, Chen Y, Cheng L (2020). Patient-derived mutations impact pathogenicity of SARS-CoV-2. medRxiv.

[5] van Dorp L, Acman M, Richard D, Shaw LP, Ford CE, Ormond L (2020). Emergence of genomic diversity and recurrent mutations in SARS-CoV-2. Infect Genet Evol.

[6] Enard D, Cai L, Gwennap C, Petrov DA (2016). Viruses are a dominant driver of protein adaptation in mammals. eLife.

[7] Banerjee A, Doxey AC, Mossman K, Irving AT (2021). Unraveling the zoonotic origin and transmission of SARS-CoV-2. Trends Ecol Evol.

[8] Andersen KG, Rambaut A, Lipkin WI, Holmes EC, Garry RF (2020). The proximal origin of SARS-CoV-2. Nat Med.

[9] Ruan Y, Wen H, He X, Wu CI (2020). A theoretical exploration of the origin and early evolution of a pandemic. Sci Bull (Beijing).

[10] Leitner T, Kumar S (2020). Where did SARS-CoV-2. Mol Biol Evol.

[11] Zhou H, Ji J, Chen X, Bi Y, Li J, Wang Q (2021). Identification of novel bat coronaviruses sheds light on the evolutionary origins of SARS-CoV-2 and related viruses. Cell.

[12] Zhou P, Yang XL, Wang XG, Hu B, Zhang L, Zhang W (2020). A pneumonia outbreak associated with a new coronavirus of probable bat origin. Nature.

[13] Liu K, Tan S, Niu S, Wang J, Wu L, Sun H (2021). Cross-species recognition of SARS-CoV-2 to bat ACE2. Proc Natl Acad Sci USA.

[14] Boni MF, Lemey P, Jiang X, Lam TTY, Perry BW, Castoe TA (2020). Evolutionary origins of the SARS-CoV-2 sarbecovirus lineage responsible for the COVID-19 pandemic. Nat Microbiol.

[15] Lytras S, Hughes J, Martin D, de Klerk A, Lourens R, Pond SLK (2021). Exploring the natural origins of SARS-CoV-2 in the light of recombination. bioRxiv.

[16] Pekar J, Worobey M, Moshiri N, Scheffler K, Wertheim JO (2021). Timing the SARS-CoV-2 index case in Hubei province. Science.

[17] Li H, Mendelsohn E, Zong C, Zhang W, Hagan E, Wang N (2019). Human-animal interactions and bat coronavirus spillover potential among rural residents in Southern China. Biosaf Health.

[18] Wang N, Li SY, Yang XL, Huang HM, Zhang YJ, Guo H (2018). Serological evidence of bat SARS-related coronavirus infection in humans, China. Virol Sin.

[19] Singh D, Yi SV (2021). On the origin and evolution of SARS-CoV-2. Exp Mol Med.

[20] Zhao J, Cui W, Tian BP (2020). The potential intermediate hosts for SARS-CoV-2. Front Microbiol.

[21] Frutos R, Serra-Cobo J, Chen T, Devaux CA (2020). COVID-19 time to exonerate the pangolin from the transmission of SARS-CoV-2 to humans. Infect Genet Evol.

[22] Wu CI, Wen H, Lu J, Su XD, Hughes AC, Zhai W (2021). On the origin of SARS-CoV-2-The blind watchmaker argument. Sci China Life Sci.

[23] Furuse Y, Oshitani H (2020). Viruses that can and cannot coexist with humans and the future of SARS-CoV-2. Front Microbiol.

[24] Korber B, Fischer WM, Gnanakaran S, Yoon H, Theiler J, Abfalterer W (2020). Tracking changes in SARS-CoV-2 spike: evidence that D614G increases infectivity of the COVID-19 virus. Cell.

[25] Zhang L, Jackson CB, Mou H, Ojha A, Peng H, Quinlan BD (2020). SARS-CoV-spike-protein D614G mutation increases virion spike density and infectivity. Nat Commun.

[26] Yurkovetskiy L, Pascal KE, Tomkins-Tinch C, Nyalile T, Wang Y, Baum A (2020). SARS-CoV-2 Spike protein variant D614G increases infectivity and retains sensitivity to antibodies that target the receptor binding domain. bioRxiv.

[27] Volz E, Hill V, McCrone JT, Price A, Jorgensen D, O'Toole Á (2021). Evaluating the effects of SARS-CoV-2 spike mutation D614G on transmissibility and pathogenicity. Cell.

[28] Ramazzotti D, Angaroni F, Maspero D, Mauri M, D'Aliberti D, Antoniotti M (2021). Large-scale analysis of synonymous viral variants reveals global adaptation of the SARS-CoV-2 to the human codon usage. bioRxiv.

[29] Peacock TP, Penrice-Randal R, Hiscox JA, Barclay WS (2021). SARS-CoV-2 one year on evidence for ongoing viral adaptation. J Gen Virol.

[30] Akther S, Bezrucenkovas E, Li L, Sulkov B, Di L, Pante D (2021). Following the trail of one million genomes: footprints of SARS-CoV-2 adaptation to humans. bioRxiv.

[31] Rochman ND, Wolf YI, Faure G, Mutz P (2021). Ongoing global and regional adaptive evolution of SARS-CoV-2. Proc Natl Acad Sci USA.

[32] Makris S, Paulsen M, Johansson C (2017). Type I interferons as regulators of lung inflammation. Front Immunol.

[33] Ye L, Schnepf D, Staeheli P (2019). Interferon- orchestrate innate and adaptive mucosal immune responses. Nat Rev Immunol.

[34] Schoggins JW (2014). Interferon-stimulated genes roles in viral pathogenesis. Curr Opin Virol.

[35] Schneider WM, Chevillotte MD, Rice CM (2014). Interferon-stimulated genes a complex web of host defenses. Annu Rev Immunol.

[36] Mattei F, Schiavoni G, Tough DF (2010). Regulation of immune cell homeostasis by type I interferons. Cytokine Growth Factor Rev.

[37] Haller O, Kochs G, Weber F (2006). The interferon response circuit induction and suppression by pathogenic viruses. Virology.

[38] Jaitin DA, Schreiber G (2007). Upregulation of a small subset of genes drives type I interferon-induced antiviral memory. J Interferon Cytokine Res.

[39] Goritzka M, Durant LR, Pereira C, Salek-Ardakani S, Openshaw PJM, Johansson C (2014). Alpha/beta interferon receptor signaling amplifies early pro-inflammatory cytokine production in the lung during respiratory syncytial virus infection. J Virol.

[40] Davidson S, Crotta S, McCabe TM, Wack A (2014). Pathogenic potential of interferon aß in acute influenza infection. Nat Commun.

[41] Goritzka M, Makris S, Kausar F, Durant LR, Pereira C, Kumagai Y (2015). Alveolar macrophage-derived type I interferons orchestrate innate immunity to RSV through recruitment of antiviral monocytes. J Exp Med.

[42] Asselin-Paturel C, Brizard G, Chemin K, Boonstra A, O'Garra A.Vicari A (2005). Type I interferon dependence of plasmacytoid dendritic cell activation and migration. J Exp Med.

[43] Le Bon A, Thompson C, Kamphuis E, Durand V, Rossman C, Kalinke U (2006). Cutting edge enhancement of antibody responses through direct stimulation of B and T cells by type I IFN. J Immunol.

[44] Channappanavar R, Fehr AR, Vijay R, Mack M, Zhao J, Meyerholz DK (2016). Dysregulated type I interferon and inflammatory monocyte-macrophage responses cause lethal pneumonia in SARS-CoV-infected mice. Cell Host Microbe.

[45] Blanco-Melo D, Nilsson-Payant BE, Liu W-C, Uhl S, Hoagland D, Møller R (2020). Imbalanced host response to SARS-CoV-2 drives development of COVID-19. Cell.

[46] Acharya D, Liu GQ, Gack MU (2020). Dysregulation of type I interferon responses in COVID-19. Nat Rev Immunol.

[47] Chu H, Chan JFW, Wang Y, Yuen TT, Chai Y, Hou Y (2020). Comparative replication and immune activation profiles of SARS-CoV-2 and SARS-CoV in human lungs an ex vivo study with implications for the pathogenesis of COVID-19. Clin Infect Dis.

[48] Hadjadj J, Yatim N, Barnabei L, Corneau A, Boussier J, Smith N (2020). Impaired type I interferon activity and exacerbated inflammatory responses in severe COVID-19 patients. Science.

[49] Ribero MS, Jouvenet N, Dreux M, Nisole S (2020). Interplay between SARS-CoV-2 and the type I interferon response. PLoS Pathog.

[50] Konno Y, Kimura I, Uriu K, Fukushi M, Irie T, Koyanagi Y (2020). SARS-CoV-2 ORF3b is a potent interferon antagonist whose activity is further increased by a naturally occurring elongation variant. Cell Rep.

[51] Yuen CK, Lam JY, Wong WM, Mak LF, Wang X, Chu H (2020). SARS-CoV-2 nsp13, nsp14, nsp15 and orf6 function as potent interferon antagonists. Emerg Microbes Infect.

[52] Lei X, Dong X, Ma R, Wang W, Xiao X, Tian Z (2020). Activation and evasion of type I interferon responses by SARS-CoV-2. Nat Commun.

[53] Miorin L, Kehrer T, Sanchez-Aparicio MT, Zhang K, Cohen P, Patel RS (2020). SARS-CoV-2 Orf6 hijacks Nup98 to block STAT nuclear import and antagonize interferon signaling. Proc Natl Acad Sci USA.

[54] Lin JW, Tang C, Wei HC, Du B, Chen C, Wang M (2021). Genomic monitoring of SARS-CoV-2 uncovers an Nsp1 deletion variant that modulates type I interferon response. Cell Host Microbe.

[55] Bar-On YM, Flamholz A, Phillips R, Milo R (2020). SARS-CoV-2 (COVID-19) by the numbers. eLife.

[56] Hoffmann M, Kleine-Weber H, Schroeder S, Krüger N, Herrier T, Erichsen S (2020). SARS-CoV-2 cell entry depends on ACE2 and TMPRSS2 and is blocked by a clinically proven protease inhibitor. Cell.

[57] Gui M, Song W, Zhou H, Xu J, Chen S, Xiang Y (2017). Cryo-electron microscopy structures of the SARS-CoV spike glycoprotein reveal a prerequisite conformational state for receptor binding. Cell Res.

[58] Shang J, Wan Y, Luo C, Ye G, Geng Q, Auerbach A (2020). Cell entry mechanisms of SARS-CoV-2. Proc Natl Acad Sci USA.

[59] Yuan Y, Cao D, Zhang Y, Ma J, Qi J, Wang Q (2017). Cryo-EM structures of MERS-CoV and SARS-CoV spike glycoproteins reveal the dynamic receptor binding domains. Nat Commun.

[60] Walls AC, Park YP, Tortorici MA, Wall A, McGuire AT, Veesler D (2020). Structure, function, and antigenicity of the SARS-CoV-2 spike glycoprotein. Cell.

[61] Wrapp D, Wang N, Corbett KS, Goldsmith JA, Hsieh C-L, Abiona O (2020). Cryo-EM structure of the 2019-nCoV spike in the prefusion conformation. Science.

[62] Du L, He Y, Zhou Y, Liu S, Zheng BJ, Jiang S (2009). The spike protein of SARS-CoV - a target for vaccine and therapeutic development. Nat Rev Microbiol.

[63] Bailey AL, Diamond MS (2021). A Crisp(r) new perspective on SARS-CoV-2 biology. Cell.

[64] Tang T, Bidon M, Jaimes JA, Whittaker GR, Daniel S (2020). Coronavirus membrane fusion mechanism offers a potential target for antiviral development. Antiviral Res.

[65] Kikkert M (2020). Innate immune evasion by human respiratory RNA viruses. J Innate Immun.

[66] Kindler E, Thiel V (2014). To sense or not to sense viral RNA - essentials of coronavirus innate immune evasion. Curr Opin Microbiol.

[67] Kindler E, Thiel V (2016). SARS-CoV and IFN too little, too late. Cell Host Microbe.

[68] Versteeg GA, Bredenbeek PJ, van den Worm SHE.Spaan WJM (2007). Group 2 coronaviruses prevent immediate early interferon induction by protection of viral RNA from host cell recognition. Virology.

[69] Klein S, Cortese M, Winter SL, Wachsmuth-Melm M, Neufeldt CJ, Cerikan B (2020). SARS-CoV-2 structure and replication characterized by in situ cryo-electron tomography. Nat Commun.

[70] Decroly E, Ferron F, Lescar J, Canard B (2012). Conventional and unconventional mechanisms for capping viral mRNA. Nat Rev Microbiol.

[71] Züst R, Cervantes-Barragan L, Habjan M, Maier R, Neuman BW, Ziebuhr J (2011). Ribose 2&apos;-O-methylation provides a molecular signature for the distinction of self and non-self mRNA dependent on the RNA sensor Mda5. Nat Immunol.

[72] Kindler E, Gil-Cruz C, Spanier J, Li Y, Wilhelm J, Rabouw HH (2017). Early endonuclease-mediated evasion of RNA sensing ensures efficient coronavirus replication. PLoS Pathog.

[73] Deng X, Hackbart M, Mettelman RC, O'Brien A.Mielech AM (2017). Coronavirus non-structural protein 15 mediates evasion of dsRNA sensors and limits apoptosis in macrophages. Proc Natl Acad Sci USA.

[74] Hackbart M, Deng X, Baker SC (2020). Coronavirus endoribonuclease targets viral polyuridine sequences to evade activating host sensors. Proc Natl Acad Sci USA.

[75] Wong HH, Sanyal S (2020). Manipulation of autophagy by (+) RNA viruses. Semin Cell Dev Biol.

[76] Yin Z, Pascual C, Klionsky DJ (2016). Autophagy machinery and regulation. Microb Cell.

[77] Koepke L, Hirschenberger M, Hayn M, Kirchhoff F, Sparrer KMj (2021). Manipulation of autophagy by SARS-CoV-2 proteins. Autophagy.

[79] Qu Y, Wang X, Zhu Y, Wang W, Wang Y, Hu G (2021). ORF3a-mediated incomplete autophagy facilitates severe acute respiratory syndrome coronavirus-2 replication. Front Cell Dev Biol.

[80] Zhang Y, Sun H, Pei R, Mao B, Zhao Z, Li H (2021). The SARS-CoV-2 protein ORF3a inhibits fusion of autophagosomes with lysosomes. Cell Discov.

[81] Samir P, Malireddi RKS, Kanneganti TD (2020). The PANoptosome a deadly protein complex driving pyroptosis, apoptosis, and necroptosis (PANoptosis). Front Cell Infect Microbiol.

[82] Christgen S, Zheng M, Kesavardhana S, Karki R, Malireddi RKS, Banoth B (2020). Identification of the PANoptosome a molecular platform triggering pyroptosis, apoptosis, and necroptosis (PANoptosis). Front Cell Infect Microbiol.

[83] Danthi P (2016). Viruses and the diversity of cell death. Annu Rev Virol.

[84] Galluzzi L, Brenner C, Morselli E, Touat Z, Kroemer G (2008). Viral control of mitochondrial apoptosis. PLoS Pathog.

[85] Richard A, Tulasne D (2012). Caspase cleavage of viral proteins, another way for viruses to make the best of apoptosis. Cell Death Dis.

[86] Upton JW, Chan FK (2014). Staying alive cell death in antiviral immunity. Mol Cell.

[87] Tao X, Hill TE, Morimoto C, Peters CJ, Ksiazek TG, Tseng CTK (2013). Bilateral entry and release of Middle East Respiratory Syndrome Coronavirus induce profound apoptosis of human bronchial epithelial cells. J Virol.

[88] Krähling V, Stein DA, Spiegel M, Weber F, Mühlberger E (2009). Severe acute respiratory syndrome coronavirus triggers apoptosis via protein kinase R but is resistant to its antiviral activity. J Virol.

[89] Ye Z, Wong CK, Li P, Xie Y (2008). A SARS-CoV protein, ORF-6, induces caspase-3 mediated, ER stress and JNK-dependent apoptosis. Biochim Biophys Acta.

[90] Sharma K, Akerström S, Sharma AK, Chow VTK, Teow S, Abrenica B (2011). SARS-CoV 9b protein diffuses into nucleus, undergoes active Crm1 mediated nucleocytoplasmic export and triggers apoptosis when retained in the nucleus. PLoS One.

[91] Lei Y, Moore CB, Liesman RM, O'Connor BP.Bergstralh DT.Chen ZJ (2009). MAVS-mediated apoptosis and its inhibition by viral proteins. PLoS One.

[92] Diemer C, Schneider M, Seebach J, Quaas J, Frösner G, Schätzl HM (2008). Cell type-specific cleavage of nucleocapsid protein by effector caspases during SARS Coronavirus infection. J Mol Biol.

[93] Freundt EC, Yu L, Goldsmith CS, Welsh S, Cheng A, Yount B (2010). The open reading frame 3a protein of severe acute respiratory syndrome-associated coronavirus promotes membrane rearrangement and cell death. J Virol.

[94] Law PTW, Wong CH, Au TCC, Chuck CP, Kong SK, Chan PKS (2005). The 3a protein of severe acute respiratory syndrome-associated coronavirus induces apoptosis in Vero E6 cells. J Gen Virol.

[95] Ren Y, Shu T, Wu D, Mu J, Wang C, Huang M (2020). The ORF3a protein of SARS-CoV-2 induces apoptosis in cells. Cell Mol Immunol.

[96] Ivanisenko NV, Seyrek K, Kolchanov NA, Ivanisenko VA, Lavrik IN (2020). The role of death domain proteins in host response upon SARS-CoV-2 infection modulation of programmed cell death and translational applications. Cell Death Discov.

[97] Kearney CJ, Martin SJ (2017). An inflammatory perspective on necroptosis. Mol Cell.

[98] Feng L, Yin YY, Liu CH, Xu KR, Li QR, Wu JR (2020). Proteome-wide data analysis reveals tissue-specific network associated with SARS-CoV-2 infection. J Mol Cell Biol.

[99] Mocarski ES, Kaiser WJ, Livingston-Rosanoff D, Upton JW, Daley-Bauer LP (2014). True grit programmed necrosis in antiviral host defense, inflammation, and immunogenicity. J Immunol.

[100] Pasparakis M, Vandenabeele P (2015). Necroptosis, and its role in inflammation. Nature.

[101] Chan FKM, Luz NF, Moriwaki K (2015). Programmed necrosis in the cross talk of cell death and inflammation. Annu Rev Immunol.

[102] Kaiser WJ, Upton JW, Mocarski ES (2013). Viral modulation of programmed necrosis. Curr Opin Virol.

[103] Li S, Zhang Y, Guan Z, Li H, Ye M, Chen X (2020). SARS-CoV-2 triggers inflammatory responses and cell death through caspase-8 activation. Signal Transduct Target Ther.

[104] Guo H, Callaway JB, Ting JP (2015). Inflammasomes mechanism of action, role in disease, and therapeutics. Nat Med.

[105] Zhao C, Zhao W (2020). NLRP3 inflammasome - A key player in antiviral responses. Front Immunol.

[106] Kesavardhana S, Kanneganti TD (2017). Mechanisms governing inflammasome activation, assembly and pyroptosis induction. Int Immunol.

[107] Yu J, Wu Y, Wang J (2017). Activation and role of NACHT, LRR, and PYD Domains-Containing Protein 3 inflammasome in RNA viral infection. Front Immunol.

[108] Shi CS, Nabar NR, Huang NN, Kehrl JH (2019). SARS-coronavirus open reading frame-8b triggers intracellular stress pathways and activates NLRP3 inflammasomes. Cell Death Discov.

[109] Siu KL, Yuen KS, Castaño-Rodriguez C, Ye ZW, Yeung ML, Fung SY (2019). Severe acute respiratory syndrome coronavirus ORF3a protein activates the NLRP3 inflammasome by promoting TRAF3-dependent ubiquitination of ASC. FASEB J.

[110] Chang YS, Ko BH, Ju JC, Chang HH, Huang SH, Lin CW (2020). SARS unique domain (SUD) of severe acute respiratory syndrome coronavirus induces NLRP3 inflammasome-dependent CXCL10-mediated pulmonary inflammation. Int J Mol Sci.

[111] Rodrigues TS, Sá KSG, Ishimoto AY, Becerra A, Oliveira S, Almeida L (2020). Inflammasome activation in COVID-19 patients. medRxiv.

[112] Yap JKY, Moriyama M, Iwasaki A (2020). Inflammasomes and pyroptosis as therapeutic targets for COVID-19. J Immunol.

[113] Kim NE, Kim DK, Song YJ (2021). SARS-CoV-2 Non-structural proteins 1 and 13 suppress caspase-1 and the NLRP3 inflammasome activation. Microorganisms.

[114] Feng Z, Diao B, Wang R, Wang G, Wang C, Tan Y (2020). The novel severe acute respiratory syndrome coronavirus 2 (SARS-CoV-2) directly decimates human spleens and lymph nodes. medRxiv.

[115] Diao B, Wang C, Tan Y, Chen X, Liu Y, Ning L (2020). Reduction and functional exhaustion of T cells in patients with coronavirus disease 2019 (COVID-19). Front Immunol.

[116] Liu J, Li S, Liu J, Liang B, Wang X, Wang H (2020). Longitudinal characteristics of lymphocyte responses and cytokine profiles in the peripheral blood of SARS-CoV-2 infected patients. EBioMedicine.

[117] Zheng M, Gao Y, Wang G, Song G, Liu S (2020). Functional exhaustion of antiviral lymphocytes in COVID-19 patients. Cell Mol Immunol.

[118] Neidleman J, Luo X, Frouard J, Xie G, Gill G, Stein ES (2020). SARS-CoV-2-specific T cells exhibit phenotypic features of helper function, lack of terminal differentiation, and high proliferation potential. Cell Rep Med.

[119] Wang F, Nie J, Wang H, Zhao Q, Xiong Y, Deng L (2020). Characteristics of peripheral lymphocyte subset alteration in COVID-19 pneumonia. J Infect Dis.

[120] Wan S, Yi Q, Fan S, Lv J, Zhang X, Guo L (2020). Characteristics of lymphocyte subsets and cytokines in peripheral blood of 123 hospitalized patients with 2019 novel coronavirus pneumonia (NCP). medRxiv.

[121] Zhang H, Kang Z, Gong H, Xu D, Wang J, Li Z (2020). The digestive system is a potential route of 2019-nCov infection: a bioinformatics analysis based on single-cell transcriptomes. bioRxiv.

[122] Xu H, Zhong L, Deng J, Peng J, Dan H, Zeng X (2020). High expression of ACE2 receptor of 2019-nCoV on the epithelial cells of oral mucosa. Int J Oral Sci.

[123] Wang K, Chen W, Zhang Z, Deng Y, Lian JQ, Du P (2020). CD147-spike protein is a novel route for SARS-CoV-2 infection to host cells. Signal Transduct Target Ther.

[124] Fenizia C, Galbiati S, Vanetti C, Vago R, Clerici M, Tacchetti C (2021). SARS-CoV-2 entry at the crossroads of CD147 and ACE2. Cells.

[125] Pontelli MC, Castro IA, Martins RB, Veras FP, La Serra L, Nascimento DC (2020). Infection of human lymphomononuclear cells by SARS-CoV-2. bioRxiv.

[126] Huang C, Wang Y, Li X, Ren L, Zhao J, Hu Y (2020). Clinical features of patients infected with 2019 novel coronavirus in Wuhan, China. Lancet.

[127] Kamphuis E, Junt T, Waibler Z, Forster R, Kalinke U (2006). Type I interferons directly regulate lymphocyte recirculation and cause transient blood lymphopenia. Blood.

[128] Shiow LR, Rosen DB, Brdickova N, Xu Y, An J, Lanier LL (2006). CD69 acts downstream of interferon-alpha/beta to inhibit S1P1 and lymphocyte egress from lymphoid organs. Nature.

[129] Kennedy DE, Knight KL (2015). Inhibition of B-lymphopoiesis by adipocytes and IL-1-producing MDSCs. J Immunol.

[130] Mehta AK, Gracias DT, Croft M (2018). TNF activity and T cells. Cytokine.

[131] Brenner D, Krammer PH, Arnold R (2008). Concepts of activated T cell death. Crit Rev Oncol Hematol;.

[132] Arnold R, Brenner D, Becker M, Frey CR, Krammer PH (2006). How T lymphocytes switch between life and death. Eur J Immunol.

[133] Liao YC, Liang WG, Chen FW, Hsu JH, Yang JJ, Chang MS (2002). IL-19 induces production of IL-6 and TNF-a and results in cell apoptosis through TNF- a. J Immunol.

[134] Morrow KN, Coopersmith CM, Ford ML (2019). IL-17, IL-27, and IL-33 a novel axis linked to immunological dysfunction during sepsis. Front Immunol.

[135] Panesar NS (2008). What caused lymphopenia in SARS and how reliable is the lymphokine status in glucocorticoid-treated patients. Med Hypotheses.

[136] Herold MJ, McPherson KG, Reichardt HM (2006). Glucocorticoids in T cell apoptosis and function. Cell Mol Life Sci.

[137] Fischer K, Hoffmann P, Voelkl S, Meidenbauer N, Ammer J, Edinger M (2007). Inhibitory effect of tumor cell-derived lactic acid on human T cells. Blood.

[138] Sanchez-Fueyo A, Markmann JF (2016). Immune exhaustion and transplantation. Amer J Transplant.

[139] Wherry EJ, Kurachi M (2015). Molecular and cellular insights into T cell exhaustion. Nat Rev Immunol.

[140] Kurachi M (2019). CD8+ T cell exhaustion. Semin Immunopathol.

[141] Pauken KE, Sammons MA, Odorizzi PM, Manne S, Godec J, Khan O (2016). Epigenetic stability of exhausted T cells limits durability of reinvigoration by PD-1 blockade. Science.

[142] Wherry EJ (2011). T cell exhaustion. Nat Immunol.

[143] Kahan SM, Wherry EJ, Zajac AJ (2015). T cell exhaustion during persistent viral infections. Virology.

[144] Bengsch B, Johnson AL, Kurachi M, Odorizzi PM, Pauken KE, Attanasio J (2016). Bioenergetic insufficiencies due to metabolic alterations regulated by the inhibitory receptor PD-1 are an early driver of CD8+ T cell exhaustion. Immunity.

[145] Cook KD, Whitmire JK (2013). The depletion of NK cells prevents T cell exhaustion to efficiently control disseminating virus infection. J Immunol.

[146] Penaloza-MacMaster P, Kamphorst AO, Wieland A, Araki K, Iyer SS, West EE (2014). Interplay between regulatory T cells and PD-1 in modulating T cell exhaustion and viral control during chronic LCMV infection. J Exp Med.

[147] Zheng HY, Zhang M, Yang CX, Zhang N, Wang XC (2020). Elevated exhaustion levels and reduced functional diversity of T cells in peripheral blood may predict severe progression in COVID-19 patients. Cell Mol Immunol.

[148] Riva G, Nasillo V, Tagliafico E, Trenti T, Luppi M (2020). COVID-19 room for treating T cell exhaustion?. Crit Care.

[149] Antonioli L, Fornai M, Blandizzi C (2020). NKG2A and COVID-19 another brick in the wall. Cell Mol Immunol.

[150] De Biasi S, Meschiari M, Gibellini L, Bellinazzi C, Borella R, Fidanza L (2020). Marked T cell activation, senescence, exhaustion and skewing towards TH17 in patients with Covid-19 pneumonia. Nat Commun.

[151] Cardone M, Yano M, Rosenberg AS, Puig M (2020). Lessons learned to date on COVID-19 hyperinflammatory syndrome considerations for interventions to mitigate SARS-CoV-2 viral infection and detrimental hyperinflammation. Front Immunol.

[152] Di Cosimo S, Malfettone A, Pérez-García JM, Llombart-Cussac A, Miceli R, Curigliano G (2020). Immune checkpoint inhibitors a physiology-driven approach to the treatment of coronavirus disease 2019. Eur J Cancer.

[153] Bouadma L, Wiedemann A, Patrier J, Surenaud M, Wicky P-H, Foucat E (2020). Immune alterations during SARS-CoV-2-related acute respiratory distress syndrome. J Clin Immunol.

[154] Marraco SAF, Neubert NJ, Verdeil G, Speiser DE (2015). Inhibitory receptors beyond T cell exhaustion. Front Immunol.

[155] Schub D, Klemis V, Schneitler S, Mihm J, Lepper PM, Wilkens H (2020). High levels of SARS-CoV-2-specific T cells with restricted functionality in severe courses of COVID-19. JCI Insight.

[156] Rogers MC, Williams JV (2019). Reining in the CD8+ T cell respiratory virus infection and PD-1-mediated T-cell impairment. PLoS Pathog.

[157] Vallbracht S, Unsold H, Ehl S (2006). Functional impairment of cytotoxic T cells in the lung airways following respiratory virus infections. Eur J Immunol.

[158] Rutigliano JA, Sharma S, Morris MY, Oguin 3rd TH, McClaren JL, Doherty PC (2014). Highly pathological influenza A virus infection is associated with augmented expression of PD-1 by functionally compromised virus-specific CD8+ T cells. J Virol.

[159] Telcian AG, Laza-Stanca V, Edwards MR, Harker JA, Wang H, Bartlett NW (2011). RSV-induced bronchial epithelial cell PD-L1 expression inhibits CD8+ T cell nonspecific antiviral activity. J Infect Dis.

[160] Loewe L, Hill WG (2010). The population genetics of mutations good, bad and indifferent. Philos Trans R Soc Lond B Biol Sci.

[161] Duffy S (2018). Why are RNA virus mutation rates so damn high. PLoS Biol.

[162] Zharikova D, Mozdzanowska K, Feng J, Zhang M, Gerhard W (2005). Influenza type A virus escape mutants emerge in vivo in the presence of antibodies to the ectodomain of Matrix Protein 2. J Virol.

[163] Bowen DG, Walker CM (2005). Mutational escape from CD8+ T cell immunity HCV evolution, from chimpanzees to man. J Exp Med.

[164] Yang ZY, Werner HC, Kong W-P, Leung K, Traggiai E, Lanzavecchia A (2005). Evasion of antibody neutralization in emerging severe acute respiratory syndrome coronaviruses. Proc Natl Acad Sci USA.

[165] Sanjuán R, Domingo-Calap P (2016). Mechanisms of viral mutation. Cell Mol Life Sci.

[166] Peck KM, Lauring AS (2018). Complexities of viral mutation rates. J Virol.

[167] Grubaugh ND, Petrone ME, Holmes EC (2020). We shouldn't worry when a virus mutates during disease outbreaks. Nat Microbiol.

[168] Smith EC, Blanc H, Vignuzzi M, Denison MR (2013). Coronaviruses lacking exoribonuclease activity are susceptible to lethal mutagenesis evidence for proofreading and potential therapeutics. PLoS Pathog.

[169] Ou J, Zhou Z, Dai R, Zhang J, Lan W, Zhao S (2020). Emergence of RBD mutations in circulating SARS-CoV-2 strains enhancing the structural stability and human ACE2 receptor affinity of the spike protein. bioRxiv.

[170] Phan T (2020). Genetic diversity and evolution of SARS-CoV-2. Infect Genet Evol.

[171] Kim SJ, Nguyen VG, Park YH, Park BK, Chung HC (2020). A novel synonymous mutation of SARS-CoV-2 Is this possible to affect their antigenicity and immunogenicity?. Vaccines.

[172] Soh SM, Kim Y, Kim C, Jang US, Lee HR (2021). The rapid adaptation of SARS-CoV-2-rise of the variants transmission and resistance. J Microbiol.

[173] Harvey WT, Carabelli AM, Jackson B, Gupta RK, Thomson EC, Harrison EM (2021). SARS-CoV-2 variants, spike mutations and immune escape. Nat Rev Microbiol.

[174] Frost SDW, Magalis BR, Pond SLK (2018). Neutral theory and rapidly evolving viral pathogens. Mol Biol Evol.

[175] Garcia-Beltran WF, Lam EC, St Denis K, Nitido AD, Garcia ZH, Hauser BM (2021). Multiple SARS-CoV-2 variants escape neutralization by vaccine-induced humoral immunity. Cell.

[176] Zhou D, Dejnirattisai W, Supasa P, Liu C, Mentzer AJ, Ginn HM (2021). Evidence of escape of SARS-CoV-2 variant B.1.351 from natural and vaccine-induced sera. Cell.

[177] Geers D, Shamier MC, Bogers S, den Hartog G, Gommers L, Nieuwkoop NN (2021). SARS-CoV-2 variants of concern partially escape humoral but not T-cell responses in COVID-19 convalescent donors and vaccinees. Sci Immunol.

[178] Hoffmann M, Arora P, Gross R, Seidel A, Hörnich BF, Hahn AS (2021). SARS-CoV-2 variants B.1.351 and P.1 escape from neutralizing antibodies. Cell.

[179] Tonkin-Hill G, Martincorena I, Amato R, Lawson ARJ, Gerstung M, Johnston I (2021). Patterns of within-host genetic diversity in. SARS-CoV-2. eLife.

[180] Lythgoe KA, Hall M, Ferretti L, de Cesare M, McIntyre-Cockett G, Trebes A (2021). SARS-CoV-2 within-host diversity and transmission. Science.

[181] Sette A, Crotty S (2021). Adaptive immunity to SARS-CoV-2 and COVID-19. Cell.

[182] Tosta E (2021). The protective immunity induced by SARS-CoV-2 infection and vaccination a critical appraisal. Explor Immunol.

[183] Forni D, Cagliani R, Pontremoli C, Mozzi A, Pozzoli U, Clerici M (2020). Antigenic variation of SARS-CoV-2 in response to immune pressure. Mol Ecol.

[184] Georgieva M, Buckee CO, Lipsitch M (2019). Models of immune selection for multi-locus antigenic diversity of pathogens. Nat Rev Immunol.

[185] Zhang C, Jin X, Chen X, Qiu L, Leng Q, Qiu T (2021). Antigenic evolution on a global scale reveals the potential natural selection of severe acute respiratory syndrome-coronavirus 2 by pre-existing cross-reactive T-cell immunity. Front Microbiol.

[186] Alisoltani A, Jaroszewski L, Iyer M, Iranzadeh A, Godzik A (2021). Increased frequency of recurrent in-frame deletions in new expanding lineages of SARS CoV-2 reflects immune selective pressure. bioRxiv.

[187] Moore JP, Offit PA (2021). SARS-CoV-2 vaccines and the growing threat of viral variants. JAMA.

[188] Andreano E, Piccini G, Licastro D, Casalino L, Johnson NV, Paciello I (2020). SARS-CoV-2 escape in vitro from a highly neutralizing COVID-19 convalescent plasma. bioRxiv.

[189] Kemp SA, Collier DA, Datir RP, Ferreira IATM, Gayed S, Jahun A (2021). SARS-CoV-2 evolution during treatment of chronic infection. Nature.

[190] Chen L, Zody MC, Di Germanio C, Martinelli R, Mediavilla JR, Cunningham MH (2021). Emergence of multiple. SARS-CoV-2 antibody escape variants in an immunocompromised host undergoing convalescent plasma treatment. mSphere.

[191] Clark SA, Clark LE, Pan J, Coscia A, McKay LGA, Shankar S (2021). SARS-CoV-2 evolution in an immunocompromised host reveals shared neutralization escape mechanisms. Cell.

[192] Moelling K (2021). Within-host and between-host evolution in SARS-CoV-2 - New variant's source. Viruses.

[193] Kustin T, Harel N, Finkel U, Perchik S, Harari S, Tahor M (2021). Evidence for increased breakthrough rates of SARS-CoV-2 variants of concern in BNT162b2-mRNA-vaccinated individuals. Nat Med.

[194] Luo H (2016). Interplay between the virus and the ubiquitin-proteasome system molecular mechanism of viral pathogenesis. Curr Opin Virol.

[195] Glickman MH, Ciechanover A (2002). The ubiquitin-proteasome proteolytic pathway destruction for the sake of construction. Physiol Rev.

[196] Isaacson MK, Ploegh HL (2009). Ubiquitination, ubiquitin-like modifiers, and deubiquitination in viral infection. Cell Host Microbe.

[197] Ratia K, Kilianski A, Baez-Santos YM, Baker SC, Mesecar A (2014). Structural basis for the ubiquitin-linkage specificity and deISGylating activity of SARS-CoV papain-like protease. PLoS Pathog.

[198] Lindner HA, Lytvyn V, Qi H, Lachance P, Ziomek E, Ménard T (2007). Selectivity in ISG15 and ubiquitin recognition by the SARS coronavirus papain-like protease. Arch Biochem Biophys.

[199] Freitas BT, Durie IA, Murray J, Longo JE, Miller HC, Crich D (2020). Characterization and noncovalent inhibition of the deubiquitinase and deISGylase activity of SARS-CoV-2 papain-like protease. ACS Infect Dis.

[200] Tiku V, Tan MW, Dikic I (2020). Mitochondrial functions in infection and immunity. Trends Cell Biol.

[201] Arnoult D, Carneiro L, Tattoli I, Girardin SE (2009). The role of mitochondria in cellular defense against microbial infection. Semin Immunol.

[202] Anand SK, Tikoo SK (2013). Viruses as modulators of mitochondrial functions. Adv Virol.

[203] Zhang L, Qin Y, Chen M (2018). Viral strategies for triggering and manipulating mitophagy. Autophagy.

[204] Wu K, Zou J, Chang HY (2020). RNA-GPS predicts SARS-CoV-2 RNA localization to host mitochondria and nucleolus. Cell Systems.

[205] Gordon DE, Jang GM, Bouhaddou M, Xu J, Obernier K, White KM (2020). A SARS-CoV-2-human protein-protein interaction map reveals drug targets and potential drug-repurposing. Nature.

[206] Singh KK, Chaubey G, Chen JY, Suravajhala P (2020). Decoding SARS-CoV-2 hijacking of host mitochondria in COVID-19 pathogenesis. Am J Physiol Cell Physiol.

[207] Tirado SMC, Yoon KJ (2003). Antibody-dependent enhancement of virus infection and disease. Viral Immunol.

[208] Wan Y, Shang J, Sun S, Tai W, Chen J, Geng Q (2020). Molecular mechanism for antibody-dependent enhancement of coronavirus entry. J Virol.

[209] Wang SF, Tseng SP, Yen CH, Yang JY, Shen CW, Chen KH (2014). Antibody-dependent SARS Coronavirus infection is mediated by antibodies against spike proteins. Biochem Biophys Res Commun.

[210] Yip MS, Leung NHL, Cheung CY, Li PH, Lee HHY, Daëron M (2014). Antibody-dependent infection of human macrophages by severe acute respiratory syndrome coronavirus. Virol J.

[211] Tetro JA (2020). Is COVID-19 receiving ADE from other coronaviruses. Microbes Infect.

[212] Peron JPS, Nakaya HI (2020). Susceptibility of the elderly to SARS-CoV-2 infection ACE-2 overexpression, shedding and antibody-dependent enhancement (ADE). Clinics.

[213] Wang J, Zand MS (2020). The potential for antibody-dependent enhancement of SARS-CoV-2 infection: translational implications for vaccine development. J Clin Transl Sci.

[214] Ricke DO, Malone RW (2020). Medical countermeasures analysis of 2019-nCoV and vaccine risks for antibody-dependent enhancement (ADE). Preprints.

[215] Iwasaki A, Yang Y (2020). The potential danger of suboptimal antibody responses in COVID-19. Nat Rev Immunol.

[216] Kulkarni R, Bramhachari P (2020). Dynamics of immune activation in viral diseases.

[217] Takada A, Kawaoka Y (2003). Antibody-dependent enhancement of viral infection molecular mechanisms and in vivo implications. Rev Med Virol.

[218] Kam YW, Kien F, Roberts A, Cheung YC, Lamirande EW, Vogel L (2007). Antibodies against trimeric S glycoprotein protect hamsters against SARS CoV challenge despite their capacity to mediate Fc gamma RII-dependent entry into B cells in vitro. Vaccine.

[219] Jaume M, Yip MS, Cheung CY, Leung HL, Li PH, Kien F (2011). Anti-severe acute respiratory syndrome coronavirus spike antibodies trigger infection of human immune cells via a pH- and cysteine protease-independent Fc R pathway. J Virol.

[220] Yilla M, Harcourt BH, Hickman CJ, McGrew M, Tamin A, Goldsmith CS (2005). SARS-coronavirus replication in human peripheral monocytes/macrophages. Virus Res.

[221] Chan PKS, Chen GG (2018). Mechanisms of lymphocyte loss in SARS coronavirus infection. Hong Kong Med J.

[222] Li L, Wo J, Shao J, Zhu H, Wu N, Li M (2003). SARS-coronavirus replicates in mononuclear cells of peripheral blood (PBMCs) from SARS patients. J Clin Virol.

[223] Nakagawa K, Lokugamage KG, Makino S (2016). Viral and cellular mRNA translation in coronavirus-infected cells. Adv Virus Res.

[224] Zou L, Ruan F, Huang M, Liang L, Huang H, Hong Z (2020). SARS-CoV-2 viral load in upper respiratory specimens of infected patients. N Engl J Med.

[225] Lee N, Chan PK, Ip M, Wong E, Ho J, Ho C (2006). Anti-SARS-CoV IgG response in relation to disease severity of severe acute respiratory syndrome. J Clin Virol.

[226] Ho MS, Chen WJ, Chen HY, Lin SF, Wang MC, Di J (2005). Neutralizing antibody response and SARS severity. Emerg Infect Dis.

[227] Forni D, Cagliani R, Clerici, Sironi M (2017). Molecular evolution of human coronavirus genomes. Trends Microbiol.

[228] Vijgen L, Keyaerts E, Moës E, Thoelen I, Wollants E, Lemey P (2005). Complete genomic sequence of human coronavirus OC43 molecular clock analysis suggests a relatively recent zoonotic coronavirus transmission event. J Virol.

[229] Graham RL, Donaldson EF, Baric RS (2013). A decade after SARS strategies for controlling emerging coronaviruses. Nat Rev Microbiol.

[230] WHO Middle East Respiratory Syndrome Coronavirus (MERS-CoV): 2019. https://www.who.int/emergencies/mers-cov/en/.

[231] Mostafa A, Kandeil A, Shehata M, El Shesheny R, Samy AK, Kayali G (2020). Middle East Respiratory Syndrome Coronavirus (MERS-CoV) state of the science. Microorganisms.

[232] WHO Summary of probable SARS cases with onset of illness from 1 November 2002 to 31 July 2003. https://www.who.int/publications/m/item/summary-of-probable-sars-cases-with-onset-of-illness-from-1-november-2002-to-31-july-2003.

[233] WHO (2004). New case of laboratory-confirmed SARS in Guangdong, China - update 5. https://www.who.int/emergencies/disease-outbreak-news/item/2004_01_31-en.

[234] Anderson RM, Fraser C, Ghani AC, Donnelly CA, Riley S, Ferguson NM (2004). Epidemiology, transmission dynamics and control of SARS the 2002-2003 epidemic. Philos Trans R Soc Lond B Biol Sci.

[235] Geoghegan JL, Holmes EC (2018). The phylogenomics of evolving virus virulence. Nat Rev Genet.

[236] Telenti A, Arvin A, Corey L, Corti D, Diamond MS, García-Sastre A (2021). Nature.

[237] Berngruber TW, Froissart R, Cjoisy M, Gandon S (2013). Evolution of virulence in emerging epidemics. PLoS Pathog.

[238] Hauser A, Counotte MJ, Margossian CC, Konstantinoudis G, Low N, Althaus CL (2020). Estimation of SARS-CoV-2 mortality during the early stages of an epidemic a modeling study in Hubei, China, and six regions in Europe. PLoS Med.

[239] Otto SP, Day T, Arino J, Colijn C, Dushoff J, Li M (2021). The origins and potential future of SARS-CoV-2 variants of concern in the evolving COVID-19 pandemic. Curr Biol.

[240] Day T, Gandon S, Lion S, Otto SP (2020). On the evolutionary epidemiology of SARS-CoV-2. Curr Biol.

[241] Miller IF, Metcalf CJE (2020). No current evidence for risk of vaccine-driven virulence evolution in SARS-CoV-2. medRxiv.

[242] Luo R, Delaunay-Moisan A, Timmis K, Danchin A (2021). SARS-CoV-2 biology and variants anticipation of viral evolution and what needs to be done. Environ Microbiol.

[243] Alizon S, Sofonea MT (2021). SARS-CoV-2 virulence evolution: avirulence theory, immunity and trade-offs. J Evol Biol.

[244] Wilder-Smith A, Chiew CJ, Lee VJ (2020). Can we contain the COVID-19 outbreak with the same measures as for SARS. Lancet Infect Dis.

[245] He D, Lui R, Wang L, Tse CK, Yang L, Stone L (2015). Global spatio-temporal patterns of influenza in the post-pandemic era. Sci Rep.

[246] Li Y, Wang X, Nair H (2020). Global seasonality of human seasonal coronaviruses a clue for postpandemic circulating season of severe acute respiratory syndrome coronavirus 2?. J Infect Dis.

[247] Kissler SM, Tedjanto C, Goldstein E, Grad YH, Lipsitch M (2020). Projecting the transmission dynamics of SARS-CoV-2 through the postpandemic period. Science.

[248] Beams AB, Bateman R, Adler FR (2021). Will SARS-CoV-2 become just another seasonal coronavirus. Viruses.

[249] Carlson CJ, Gomez ACR, Bansal S, Ryan SJ (2021). Misconceptions about weather and seasonality must not misguide COVID-19 response. Nat Commun.

[250] Gilbert SF, Sapp J, Tauber AI (2012). A symbiotic view of life we have never been individuals. Q Rev Biol.

[251] Gilbert SF, Rosenberg E, Zilber-Rosenberg, Gissis SB, Lamm E, Shavit A (2018). Landscapes of collectivity in the life sciences.

[252] Wang Y, Zhu N, Li YU, Lu R, Wang H, Liu G (2016). Metagenomic analysis of viral genetic diversity in respiratory samples from children with severe acute respiratory infection in China. Clin Microbiol Infect.

[253] Taboada B, Espinoza MA, Isa P, Aponte FE, Arias-Ortiz MA, Monge-Martínez J (2014). Is there still room for novel viral pathogens in pediatric respiratory tract infections. PLoS One.

[254] Lysholm F, Wetterbom A, Lindau C, Darban H, Bjerkner A, Fahlander K (2012). Characterization of the viral microbiome in patients with severe lower respiratory tract infections, using metagenomic sequencing. PLoS One.

[255] Graf EH, Simmon KE, Tardif KD, Hymas W, Flygare S, Eilbeck K (2016). Unbiased detection of respiratory viruses by use of RNA sequencing-based metagenomics a systematic comparison to a commercial PCR panel. J Clin Microbiol.

[256] Thorburn F, Bennett S, Modha S, Murdoch D, Gunson R, Murcia PR (2015). The use of next generation sequencing in the diagnosis and typing of respiratory infections. J Clin Virol.

[257] Song G, He WT, Callaghan S, Anzanello F, Huang D, Ricketts J (2021). Cross-reactive serum and memory B-cell responses to spike protein in SARS-CoV-2 and endemic coronavirus infection. Nat Commun.

[258] Sokal A, Chappert P, Barba-Spaeth G, Roeser A, Fourati S, Azzaoui I (2021). Maturation and persistence of the anti-SARS-CoV-2 memory B cell response. Cell.

[259] WHO (2020). Disease Outbreak News. SARS-CoV-2 Variants. https://www.who.int/emergencies/disease-outbreak-news/item/2020-DON305.

[260] Zhan SH, Deverman BE, Chan YA (2020). SARS-CoV-2 is well adapted for humans. What does this mean for re-emergence?. bioRxiv.

